# Stability Indicating LC-Method for Estimation of Paracetamol and Lornoxicam in Combined Dosage Form

**DOI:** 10.3797/scipharm.1012-03

**Published:** 2011-01-20

**Authors:** Dimal A. Shah, Neel J. Patel, Sunil L. Baldania, Usman K. Chhalotiya, Kashyap K. Bhatt

**Affiliations:** 1 Indukaka Ipcowala College of Pharmacy, Beyond GIDC, P.B. No. 53, Vitthal Udyognagar-388 121, Gujarat, India; 2 A. R. College of Pharmacy, P. Box No. 19, Vallabh Vidyanagar-388 120, Gujarat, India

**Keywords:** RP-HPLC, Validation, Simultaneous determination, Degradation

## Abstract

A simple, specific and stability indicating reversed phase high performance liquid chromatographic method was developed for the simultaneous determination of paracetamol and lornoxicam in tablet dosage form. A Brownlee C-18, 5 μm column having 250×4.6 mm i.d. in isocratic mode, with mobile phase containing 0.05 M potassium dihydrogen phosphate:methanol (40:60, v/v) was used. The flow rate was 1.0 ml/min and effluents were monitored at 266 nm. The retention times of paracetamol and lornoxicam were 2.7 min and 5.1 min, respectively. The linearity for paracetamol and lornoxicam were in the range of 5–200 μg/ml and 0.08–20 μg/ml, respectively. Paracetamol and lornoxicam stock solutions were subjected to acid and alkali hydrolysis, chemical oxidation and dry heat degradation. The proposed method was validated and successfully applied to the estimation of paracetamol and lornoxicam in combined tablet dosage form.

## Introduction

Lornoxicam (LRN) is chemically 6-chloro-4-hydroxy-2-methyl-*N*-(pyridin-2-yl)-2*H*-thieno[2,3-*e*][1,2]thiazine-3-carboxamide 1,1-dioxide. Lornoxicam is non steroidal anti-inflammatory drug and it inhibits prostaglandin biosynthesis by blocking the enzyme cyclooxygenase. Unlike some NSAIDs, Lornoxicam do not inhibit 5-lipoxygenase activity and thus do not inhibit leukotriene synthesis [1]. Paracetamol (PCM) is chemically 4-hydroxy-acetanilide. PCM is a weak inhibitor of peripheral cyclooxygenase and its analgesic effects may arise from inhibition of prostanoid synthesis in the CNS. The antipyretic effects of PCM are due to its action at the level of the hypothalamus to reduce pyrogen-initiated alterations in body temperature by inhibiting prostaglandin synthesis [2, 3]. The combination dosage form of paracetamol and lornoxicam is available in the market and it is indicated in the treatment of rheumatism and in reduction of post operative pain.

Literature survey revealed that liquid chromatographic (LC) [4, 5], LC-electrospray tendem mass spectrometry [6, 7] methods have been reported for the estimation of lornoxicam. Paracetamol is official in Indian Pharmacopoeia and British Pharmacopoeia. Different LC methods have been reported for the estimation of PCM [3, 8]. Literature survey revealed no method reported for the estimation of PCM and LRN in combined dosage form. Present study involves development of a stability liquid chromatographic method for the estimation of LRN and PCM in combination dosage form.

## Results and Discussion

### Optimization of mobile phase:

**Fig. 1.**
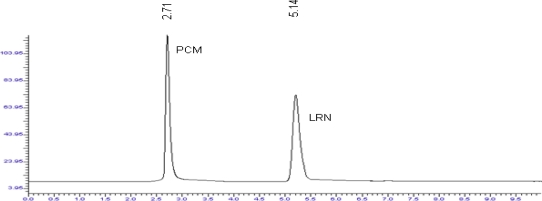
Liquid chromatogram of PCM and LRN

Optimization of mobile phase was performed based on resolution of the drugs and degradation products, asymmetric factor and theoretical plates obtained for LRN and PCM. The mobile phase consisting of 0.05 M potassium dihydrogen phosphate: methanol (40:60, v/v) was selected which gave sharp, well-resolved peaks for LRN and PCM (fig. 1).

The retention times for PCM and LRN were 2.7 and 5.1 min, respectively. The asymmetric factors for LRN and PCM were 1.2 and 1.4, respectively. UV overlaid spectra of LRN and PCM showed that all the drugs absorbed appreciably at 266 nm, so the same was selected as the detection wavelength during the studies.

### Validation:

The calibration curve was found to be linear over the range of 0.08–20 μg/ml for LRN and 5–200 μg/ml for PCM. The data of regression analysis of the calibration curves are shown in table 1.

The accuracy of the method was determined by calculating recoveries of LRN and PCM by method of standard additions. The recoveries obtained were 97.28–100.34% for LRN and 98.24–100.07% for PCM, respectively. The high values indicate that the method is accurate. Instrument precision was determined by performing injection repeatability test and the RSD values for LRN and PCM were found to be 0.61% and 0.02%, respectively. The intra-day and inter-day precision studies were carried out. For the intra-day study RSD values were found to be 0.07–0.88% for LRN and 0.05–1.12% for PCM and for inter-day precision study RSD values were found to be 0.09–1.95% for LRN and 0.07–1.45% for PCM, respectively. The low RSD values indicate that the method is precise. The detection limits for LRN and PCM were 0.007 μg/ml and 0.54 μg/ml, respectively, while quantitation limits were 0.02 μg/ml and 1.59 μg/ml, respectively. The above data shows that a nanogram quantity of both the drugs can be accurately and precisely determined. The validation parameters are summarized in table 2 and the system suitability test parameters are shown in table 3.

Robustness of the method was studied by changing the flow rate of the mobile phase from 1ml/min to 0.8 ml/min and 1.2 ml/min. Using 1.2 ml/min flow rate, retention time for LRN and PCM were observed to be 4.9 and 2.6 min, respectively and with 0.8 ml/min flow rate, retention times for LRN and PCM were observed to be 5.4 and 2.9 min, respectively without affecting the resolution of the drugs. When mobile phase composition was changed to 0.05 M potassium dihydrogen phosphate: methanol (35:65, v/v) by increasing percentage of methanol, the retention time for LRN and PCM were observed to be 4.9 and 2.7 min, respectively. The solution stability study revealed that LRN and PCM solutions were stable for 24 h with out detectable degradation and the percentage recovery of both the drugs were found to be more than 97%.

### Forced degradation study:

**Fig. 2.**
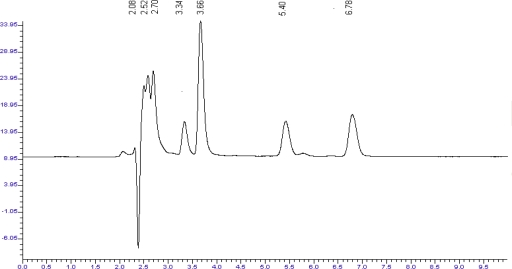
Chromatogram of base treated LRN

**Fig. 3.**
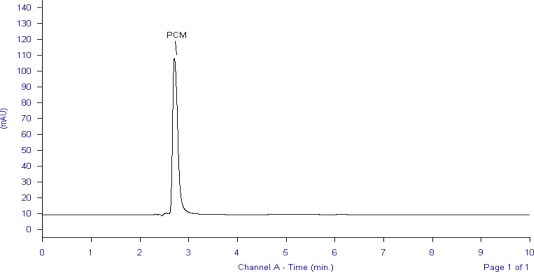
Chromatogram of base treated PCM

Forced degradation study was carried out by subjecting both the drugs to acid and alkali hydrolysis, chemical oxidation and dry heat degradation conditions. The chromatograms of base degraded sample showed degradation product peaks at retention time (RT) 3.4, 3.7, 5.5 and 6.9 min for LRN and PCM was found to be stable to base degradation (fig. 2, 3).

**Fig. 4.**
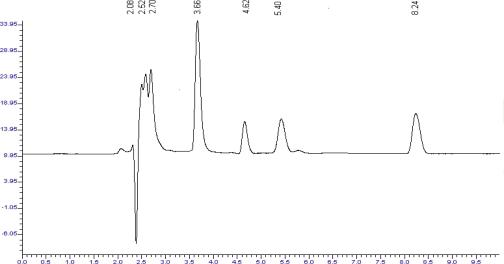
Chromatogram of acid treated LRN

**Fig. 5.**
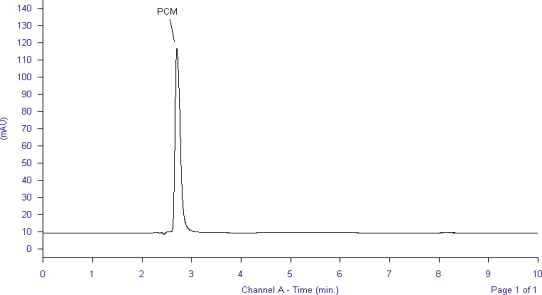
Chromatogram of acid treated PCM

The peaks of the degradation products were well resolved from the drug peaks. The chromatograms of acid degraded sample showed degradation product peaks at retention time (RT) 3.7, 4.7, 5.5 and 8.3 min for LRN and PCM was found to be stable to acid degradation (fig. 4, 5).

Both LRN and PCm were found to be stable to both oxidative stress degradation and dry heat degradation.

The degradation study thereby indicated that LRN was stable to dry heat and chemical oxidation study while it was susceptible to base and acid hydrolysis. PCM was found to be highly stable molecule which do not underwent degradation in the proposed conditions (table 4).

### Analysis of marketed formulation:

The proposed method was applied to the determination of LRN and PCM in their combined dosage form (Tablet A). The results for LRN and PCM were comparable with the corresponding labeled amounts (Table 5).

## Experimental

The liquid chromatographic system of perkin elmer series 200 containing quaternary gradient pump, variable wavelength programmable UV/Vis detector and rheodyne injector with 20 μl fixed loop was used. A Brownlee C18 column with 250×4.6 mm i.d. and 5 μm particle size was used. Analytically pure PCM was procured as gift sample from M/s Baroque Pharmaceutical Ltd., (Khambhat, India). LRN was procured as gift sample from M/s Zydus Cadila Pvt. Ltd., (Ahmedabad, India). Methanol, water (E. Merck, Mumbai, India) were of LC grade, while potassium dihydrogen phosphate (S.D. fine chemicals, Mumbai, India) was of analytical grade used for the preparation of mobile phase. Tablet formulation A (LORNASAFE-PLUS, Mankind Pharmaceutical Ltd., India) containing labeled amount of 8 mg of lornoxicam and 500 mg of paracetamol were purchased from local market

### Preparation of mobile phase and stock solution

Mobile phase was prepared by accurately weighing 6.8 g of potassium dihydrogen phosphate and dissolving in 1000 ml of water. Later 400 ml of this solution was mixed with 600 ml of methanol. The solution was filtered with Whatman filter paper (0.45 μm). The solution was sonicated for 15 min for degassing prior to use.

Stock solutions were prepared by accurately weighing 25 mg each of PCM and LRN and transferring to two separate 25 ml volumetric flasks containing 10 ml of methanol. The flasks were sonicated for 10 minutes to dissolve the solids. Volumes were made up to the mark with methanol, which gave 1000 μg/ml of both the drugs. Aliquots from the stock solutions were appropriately diluted with mobile phase to obtain working standards of 100 μg/ml of each drug.

### Chromatographic conditions

A reversed phase C_18_ column (Brownlee) equilibrated with mobile phase comprising of methanol: 0.05M potassium dihydrogen phosphate (60:40, v/v) was used. Mobile phase flow rate was maintained at 1 ml/ min and eluent were monitored at 266 nm. A 20 μL of sample was injected using a fixed loop, and the total run time was 10 min. All the chromatographic separations were carried out at controlled room temperature (20–25°C).

### Calibration curves for PCM and LRN

Appropriate aliquots of PCM working standard solution were taken in different 10 ml volumetric flasks. Appropriate aliquots of LRN working standard solution were added to the same flasks. The volumes were made up to the mark with mobile phase to obtain final concentrations of 5, 25, 50, 100, 150 and 200 µg/ml of PCM and 0.08, 0.4, 2, 10, 15 and 20 µg/ml of LRN, respectively. The solutions were injected using a 20 μL fixed loop system and chromatograms were recorded. Calibration curves were constructed by plotting peak area versus concentrations of the drug and regression equations were computed for PCM and LRN.

### Analysis of Marketed Formulations

Twenty tablets were weighed accurately and finely powdered. Tablet powder equivalent to 500mg PCM (and 8 mg of LRN) was taken in 50 ml volumetric flask. A few ml (20 ml) of methanol was added to the above flask and the flask was sonicated for 15 minutes. The solution was filtered in another 50 ml volumetric flask using Whatman filter paper and volume was made up to the mark with the same solvent.

Appropriate volume of the aliquot was transferred to a 10 ml volumetric flask and the volume was made up to the mark with the mobile phase to obtain a solution containing 100 μg/ml of PCM and 1.6 μg/ml of LRN. The solution was sonicated for 10 min. It was injected as per the above chromatographic conditions and peak area was recorded. The quantifications were carried out by keeping these values to the straight line equation of calibration curve.

### Validation

The method was validated for accuracy, precision, specificity, detection limit, quantitation limit and robustness. The accuracy of the method was determined by calculating recoveries of LRN and PCM by method of standard additions. Known amount of LRN (0, 1, 5, 10 μg/mμl) and PCM (0, 10, 50, 100 μg/ml) were added to a pre quantified sample solutions and the amount of PCM and LRN were estimated by measuring the peak area and by fitting these values to the straight-line equation of calibration curve.

The instrument precision was evaluated by injecting the solution containing LRN (2 μg/ml) and PCM (100 μg/ml) six times repeatedly and peak area was measured. The results are reported in terms of relative standard deviation. The intra-day and inter-day precision study of LRN and PCM was carried out by estimating the corresponding responses 6 times on the same day and on 3 different days (first, second and third day) for 3 different concentrations of LRN (0.08, 2, 20 μg/ml) and PCM (5, 100, 200 μg/ml), and the results are reported in terms of relative standard deviation (RSD). The specificity was estimated by spiking commonly used excipient (starch, talc and magnesium stearate) into a pre weighed quantity of drug. The chromatogram was taken by appropriate dilutions and the quantities of drugs were determined.

The detection limit is defined as the lowest concentration of an analyte that can reliably be differentiated from background levels. Limit of quantification of an individual analytical procedure is the lowest amount of analyte that can be quantitatively determined with suitable precision and accuracy. LOD and LOQ were calculated using following equation as per ICH guidelines. LOD = 3.3 ×σ /S and LOQ = 10 ×σ /S, where σ is the standard deviation of y-intercepts of regression lines and S is the slope of the calibration curve.

Robustness of the method was studied by deliberately changing the experimental conditions like flow rate, percentage of organic phase and also by observing the stability of the sample solution at 25 ± 2° for 24 h. The sample solution was assayed at every 6 h interval up to 24 h.

### Forced degradation study

Stress degradation study using acid and alkali hydrolysis, chemical oxidation and dry heat degradation was carried out and interference of the degradation products were investigated. LRN and PCM were weighed (25 mg each) and transferred to two separate 25 ml volumetric flasks and diluted up to the mark with mobile phase. These stock solutions were used for forced degradation studies.

Forced degradation in basic media was performed by taking 2.5 ml stock solutions of LRN and PCM (1000 μg/ml) in two different 25 ml volumetric flasks and 5 ml of 1 N NaOH was added. Similarly, 2.5 ml aliquots of stock solutions of LRN and PCM were taken in same 25 ml volumetric flask and 5 ml 1 N NaOH was added. All the flasks were stored at room temperature for 24 hrs. Solutions were neutralized with acid using pH meter and suitably diluted with mobile phase to obtain final concentration of 10 μg/ ml of PCM and LRN separately and in the mixture. Similarly, forced degradation in acidic medium was performed using 1 N HCl.

To perform oxidative stress degradation, appropriate aliquots of stock solutions of LRN and PCM (1000 μg/ml) were taken in two different 25 ml volumetric flasks and 5 ml of 3% hydrogen peroxide was added. Similarly, appropriate aliquots of stock solutions of PCM and LRN were taken in same 25 ml volumetric flaks and 5 ml 3% hydrogen peroxide was added. All the mixtures were stored at room temperature for 24 hrs. Solutions were diluted with mobile phase to obtain final concentration of 10 μg/ ml of PCM and LRN separately and in mixture.

To study dry heat degradation, solid drugs were exposed in oven at 80 ° for 2 h. After 2 h of heating 25 mg each of LRN and PCM were weighed and transferred to two separate volumetric flasks (25 ml) and diluted up to the mark with the mobile phase. Solutions were further diluted by taking appropriate aliquots in different 10 ml volumetric flasks to obtain final concentration of 10 μg/ml of LRN and PCM.

All the reaction solutions were injected in the liquid chromatographic system and chromatograms were recorded.

## Figures and Tables

**Tab. 1.** Regression analysis of the calibration curve for the proposed method

**Parameters**	**LRN**	**PCM**
Linearity range (μg/ml)	0.08–20	5–200
Slope	61529	61190
Standard deviation of slope	45.288	47.69
Intercept	1097	809.6
Standard deviation of intercept	285.89	298.038
Correlation coefficient	0.9980	0.9950

**Tab. 2.** Summary of validation parameters

**Parameters**	**LRN**	**PCM**
Detection limit (μg/ml)	0.007	0.54
Quantitation limit (μg/ml)	0.02	1.59
Accuracy(%)	97.28–100.34%	98.24–100.07%
Precision (RSD^a^,%)		
Intra-day precision (n=3)	0.07–0.88%	0.05–0.12%
Inter-day precision (n=3)	0.09–1.95%	0.07–1.45%
Instrument precision (RSD^a^)	0.61%	0.02%
Robustness	97.12–100.56%	98.45–99.18%

a RSD is relative standard deviation and ‘n’ is number of determinations

**Tab. 3.** System suitability test parameters for the proposed method

**System suitability parameters**	**LRN**	**PCM**
Retention time	5.1 min	2.7 min
Theoratical plates/ meter	5417	4349
Assymetric factor	1.2	1.4
Resolution	11	–

**Tab. 4.** Forced degradation study of LRN and PCM for the proposed method.

**Condition**	**Time (h)**	**Recovery (%)**		**Retention time of degradation products**	

		**PCM**	**LRN**	**PCM**	**LRN**
Base 1 N NaOH	24	97.56	32.16	–	3.4, 3.7, 5.5 and 6.9
Acid 1 N HCl	24	98.24	30.62	–	3.7, 4.7, 5.5 and 8.3
3% hydrogen peroxide	24	99.45	97.51	–	–
Dry heat^b^	2	97.92	95.62	–	–

b Samples were heated at 80° for specified period of time.

**Tab. 5.** Assay results of tablet dosage form using proposed method

**Formulation**	**Labelled Amount (mg)**		**% Recovery c**	

	**PCM**	**LRN**	**PCM**	**LRN**
A	500	8	100.01 ± 0.72	98.56 ± 0.37

c mean value ± standard deviation of three determinations; Tablet formulation A is LORNASAFE-PLUS (Mankind Pharmaceutical Ltd., India) containing labeled amount of 500 mg paracetamol and 8 mg of lornoxicam.
